# The influence of perceived need support in physical education on adolescents’ extracurricular physical activity: a cross- contextual perspective based on self-determination theory and prospect theory

**DOI:** 10.3389/fpsyg.2026.1888982

**Published:** 2026-10-08

**Authors:** Jing Bai, Linlin Li, Zheyu Zhang

**Affiliations:** 1 School of General Education, Ningxia Vocational and Technical University, Yinchuan, China; 2 School of General Education, Chongqing Jianzhu College, Chongqing, China; 3 School of Marxism, Central University of Finance and Economics, Beijing, China; 4 School of Marxism, Xizang University, Lhasa, China

**Keywords:** extracurricular physical activity, intrinsic motivation in physical education, loss aversion, perceived benefits of sports participation, perceived need support

## Abstract

**Objective:**

In research on adolescent physical activity, understanding how positive psychological experiences in physical education classes can be transformed into sustained participation in extracurricular physical activity has become a critical issue for bridging classroom instruction and out-of-class physical activity. Drawing on self-determination theory, the trans-contextual model, and prospect theory, the present study examines the mechanisms through which adolescents’ perceived need support in physical education classes influences their sustained participation in extracurricular physical activity.

**Methods:**

A two-wave longitudinal design was adopted, and data were collected from 597 Chinese secondary school students. Mediation effects were estimated using the product-of-coefficients approach combined with the bias-corrected Monte Carlo bootstrapping method.

**Results:**

The mediating effect through perceived benefits of sports participation was significant (*p* < 0.001). The mediating effect through loss aversion was also significant (*p* < 0.001).

**Conclusion:**

Adolescents’ perceived need support in physical education classes can promote their sustained participation in extracurricular physical activity through the sequential effects of intrinsic motivation in physical education (IMPE) and intrinsic motivation in extracurricular physical activity (IMECPA), as well as through the perceived benefits of sports participation and loss aversion elicited by extracurricular physical activity.

## 1 Introduction

Regular participation in physical activity provides numerous benefits for adolescents, including promoting physical development, enhancing physical fitness, improving mental health, and strengthening self-confidence (Eime et al., 2013; Agata and Monyeki, 2018). Despite the well-documented benefits of physical activity, physical activity levels among adolescents worldwide remain generally low, with more than 80% of adolescents failing to meet the recommended physical activity guidelines (Guthold et al., 2020). The situation among Chinese adolescents is similarly concerning, as only 34.1% of adolescents meet the standards recommended by the World Health Organization (Zhu et al., 2019). Against this background, school physical education classes, as the primary platform for adolescents’ physical activity, are responsible not only for instruction in physical education knowledge and motor skills but also for ensuring that students engage in a sufficient amount of physical activity. However, within the context of the Chinese school curriculum, the core emphasis of physical education classes remains on knowledge instruction and skill acquisition rather than on increasing students’ physical activity levels. Under these circumstances, students’ actual physical activity levels in physical education classes often show substantial variability and are relatively low overall, making it difficult for physical education classes alone to adequately meet adolescents’ physical activity needs (Stratton et al., 2008; Tapia-Serrano et al., 2023). Therefore, enhancing adolescents’ physical activity levels requires not only the integration of physical education classes with extracurricular physical activity but also a deeper understanding of the psychological mechanisms underlying the formation and maintenance of adolescents’ physical activity.

Self-determination theory emphasizes that when individuals’ basic psychological needs are satisfied, their intrinsic motivation is more likely to be effectively activated (Deci and Ryan, 2000). The trans-contextual model further indicates that intrinsic motivation developed in physical education classes can be transferred to extracurricular contexts, thereby promoting participation in extracurricular physical activity (Hagger et al., 2003, 2005). However, the emergence and transfer of motivation alone cannot fully explain the maintenance of physical activity. According to prospect theory, when adolescents participate in extracurricular physical activity, they may develop perceived benefits of extracurricular physical activity as a result of positive physical and psychological experiences, or experience loss aversion due to an unwillingness to lose the beneficial outcomes of extracurricular physical activity they have already attained. These two psychological mechanisms, jointly driven by the pursuit of gains and the avoidance of losses, contribute to adolescents’ sustained participation in extracurricular physical activity (Kahneman and Tversky, 1979).

Existing research has extensively examined the factors influencing extracurricular physical activity among Chinese adolescents; however, several limitations remain. First, although the trans-contextual model has been proposed to explain the transfer of motivation from physical education classes to extracurricular physical activity, much of the early empirical evidence was obtained from students living in countries such as the United Kingdom, Singapore, and Estonia (Hagger et al., 2003, 2005, 2009). Consequently, the applicability of the model to adolescents within the sociocultural and educational context of mainland China requires further systematic examination. Second, although concepts from behavioral economics have gradually been introduced into the field of health behavior research, the application of the concepts of perceived benefits of sports participation and loss aversion in physical activity research remains limited, and there is a lack of systematic explanation for adolescents’ sustained participation in extracurricular physical activity from a dual perspective integrating positive incentives and loss avoidance. Based on these research gaps, the present study integrates self-determination theory, the trans-contextual model, and prospect theory to construct a multi-theoretical integrative research model (see Figure 1), and systematically elucidates the formation and maintenance mechanisms of adolescents’ extracurricular physical activity from multiple perspectives, including intrinsic motivation formation, contextual transfer, and decision-making psychological processes.

**FIGURE 1:**
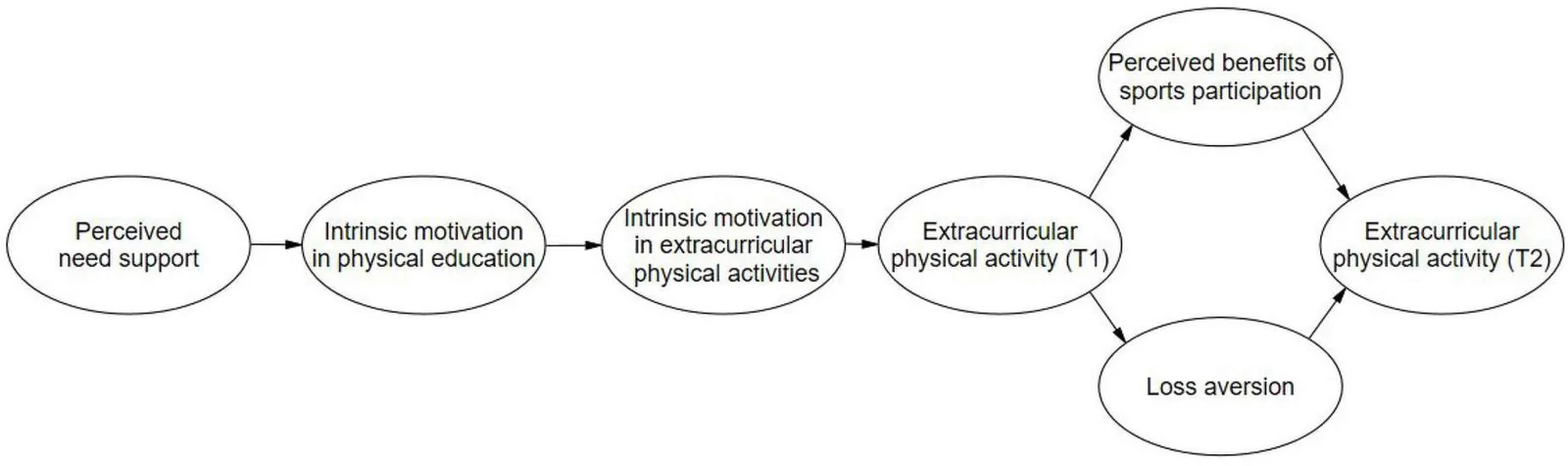
Research model.

### 1.1 The mediating role of IMPE between perceived need support and IMECPA

According to self-determination theory, intrinsic motivation refers to an individual’s psychological tendency to engage in a behavior voluntarily in the absence of external rewards, driven by interest, enjoyment, or a sense of personal value, and is considered the most ideal and enduring form of motivation. In contemporary educational research, learning motivation is regarded as a key factor influencing classroom engagement and learning outcomes (Sun, 2016). Within the framework of self-determination theory, autonomy, competence, and relatedness constitute the three basic psychological needs underlying the formation and maintenance of intrinsic motivation (Deci and Ryan, 2000). In physical education classes, when students perceive that teachers provide sufficient support for needs for autonomy, competence, and relatedness, their corresponding basic psychological needs are satisfied, thereby enhancing students’ intrinsic motivation.

In the context of physical education instruction, autonomy support typically refers to the extent to which teachers provide students with appropriate opportunities for choice and decision-making during the teaching process, thereby enabling students to experience autonomy or a sense of agency in learning activities (Xiang et al., 2011). Research has shown that students’ perceptions of teachers’ autonomy support can significantly predict classroom motivation (Black and Deci, 2000; Shen et al., 2009; Hagger et al., 2009). According to self-determination theory, the need for autonomy is one of individuals’ basic psychological needs. When students’ need for autonomy is satisfied, external instructional demands are more likely to be internalized by students, thereby fostering intrinsic motivation. In the highly interactive context of physical education classes, which emphasizes physical engagement and immediate feedback, autonomy support helps reduce students’ resistance to external control and enhances their recognition of the value of learning activities, thereby promoting the emergence and maintenance of intrinsic motivation (Chang et al., 2016). Accordingly, fostering an autonomy-supportive classroom climate in physical education not only satisfies students’ need for autonomy but also facilitates the development of intrinsic motivation, thereby enhancing classroom engagement and learning outcomes.

In physical education classes, competence support refers to students’ perceptions that they possess the ability to master learning content and achieve learning goals during the learning process (Xiang et al., 2011). Self-determination theory posits that the need for competence plays a crucial role in the development of individual motivation. When physical education classes provide students with clear learning goals, appropriately challenging tasks, and positive feedback, students are more likely to experience a sense of competence and achievement in learning activities, thereby enhancing intrinsic motivation. Relevant research has suggested that physical education curricula should prioritize the cultivation of students’ sense of competence (Sun and Chen, 2010), emphasizing not only the development of sports knowledge and physical skills but also the cultivation of interest in physical activity, attitudes, and values (Chen and Ennis, 2004). Further empirical evidence has also demonstrated that the higher individuals’ perceived competence in a given activity, the higher their level of intrinsic motivation (Ntoumanis, 2001).

Relatedness support in physical education classes refers to students’ perceptions of positive social connections with teachers, peers, or the group within the classroom context, through which they experience feelings of acceptance, respect, and a sense of belonging (Xiang et al., 2011). Relevant research has indicated that the need for relatedness constitutes a fundamental prerequisite for effective learning (Sun and Chen, 2010). When students perceive care from teachers and acceptance from peers in physical education classes, they are more likely to actively engage in classroom activities and cooperative learning, and such sustained interactions facilitate the activation and maintenance of IMPE. Further empirical evidence has shown that higher levels of relatedness support contribute to enhanced classroom engagement, learning persistence, and intrinsic motivation, thereby promoting the sustained development of students’ learning behaviors in physical education (Standage et al., 2005).

Within the Chinese physical education system, physical education classes and extracurricular physical activity constitute the primary contexts in which adolescents participate in physical activity. According to the trans-contextual model, need support and positive experiences perceived by students in physical education classes not only facilitate the development of higher levels of intrinsic motivation in the classroom but can also be transferred to extracurricular physical activity through psychological transfer mechanisms, thereby encouraging students to engage voluntarily in physical activity during their leisure time (Hagger et al., 2003; Barkoukis et al., 2010). Given the similarities between physical education classes and extracurricular physical activity in terms of activity content, motor skill application, and behavioral goals, students’ interest in physical activity and enjoyment developed in physical education classes are more likely to transfer to extracurricular contexts, thereby fostering their intrinsic motivation to participate voluntarily in extracurricular physical activity (Cox et al., 2008). However, cross-cultural research indicates that the applicability of this model varies across countries. Empirical evidence from Hagger et al. (2005) demonstrated that the model exhibited good fit in samples from the United Kingdom, Greece, and Singapore, whereas some pathways were not significant in the Polish sample. Moreover, the significance and strength of the pathways differed across countries. These findings suggest that although the trans-contextual model demonstrates considerable generalizability and structural stability across cultures, the specific mechanisms underlying its pathways may be influenced by factors such as cultural differences, educational environments, or social norms. In summary, intrinsic motivation developed in physical education classes can influence students’ motivation and behavior in extracurricular physical activity through cross-contextual transfer; however, the applicability of this model within the Chinese context requires further empirical validation.

### 1.2 The role of perceived benefits of sports participation in sustained participation in extracurricular physical activity

The perceived benefits of sports participation may play a mediating role in the relationship between participation in extracurricular physical activity and sustained participation in extracurricular physical activity. In the present study, perceived benefits of sports participation are defined as students’ subjective perceptions of the positive experiences and intrinsic gains derived from participation in extracurricular physical activity. This concept encompasses not only physiological aspects, such as improvements in physical fitness and physical appearance, but also psychological and social dimensions, including the promotion of mental health and the enhancement of interpersonal relationships. A substantial body of research has demonstrated that extracurricular physical activity plays an important role in promoting adolescents’ physical and mental health, enhancing physical fitness, and alleviating psychological stress (Guilmette et al., 2019; Tapia-Serrano et al., 2023; Aniśko et al., 2025). Specifically, at the physical level, participation in extracurricular physical activity can improve adolescents’ physical fitness and motor competence (Ara et al., 2004; Aniśko et al., 2025). At the psychological level, sports participation has been shown to facilitate emotional well-being (Guilmette et al., 2019), alleviate social anxiety (Dimech and Seiler, 2010), and significantly enhance subjective well-being (Li et al., 2024). Finally, at the social level, extracurricular physical activity contributes to the development of adolescents’ interpersonal skills (Ivaniushina and Zapletina, 2015).

The perceived benefits of sports participation are derived not only from individuals’ own positive exercise experiences but also from cognitive processes involved in observing others receive positive feedback. When adolescents perceive that physical activity can generate tangible benefits, they are more likely to develop long-term and stable exercise habits. Previous research has indicated that individuals who engage in regular exercise are more likely to experience the positive effects of physical activity, and that higher levels of perceived benefits of sports participation are associated with greater exercise frequency (Grubbs and Carter, 2002). During sports participation, individuals who possess a stronger sense of competence and experience more positive outcomes tend to demonstrate higher levels of engagement, lower levels of boredom, and stronger intentions to continue exercising (Ntoumanis, 2001).

Drawing on self-determination theory and the trans-contextual model, when students perceive classroom need support from teachers, their intrinsic motivation is activated and subsequently transferred to extracurricular contexts, thereby promoting participation in extracurricular physical activity (T1). Subsequently, individuals develop perceived benefits of sports participation through exercise, which further facilitate sustained participation in extracurricular physical activity. Accordingly, the following hypothesis is proposed:

H1: Adolescents’ positive perceptions of teachers’ need support can have a significant positive effect on their sustained participation in extracurricular physical activity through the sequential mediating effects of IMPE and IMECPA, as well as through the perceived benefits of sports participation associated with early extracurricular physical activity (T1).

### 1.3 The role of loss aversion in sustained engagement in extracurricular physical activity

The term loss aversion originates from behavioral economics and represents a core concept of prospect theory (Kahneman and Tversky, 1979). According to this theory, in decision-making contexts, individuals respond more strongly to losses than to gains of equal magnitude, such that the negative impact of losses is typically greater than the positive impact associated with equivalent gains (Kahneman and Tversky, 1979). Research has shown that although loss aversion varies significantly across different populations, it exhibits relative stability at the individual level (Brown et al., 2014). In the present study, loss aversion is conceptualized as individuals’ tendency to avoid the potential negative consequences resulting from non-participation in physical activity. Specifically, students’ participation in extracurricular physical activity is not driven solely by interest in the activity itself, but also by a motivation to avoid potential risks such as deterioration in body shape, declines in physical fitness, and increased psychological stress.

Prospect theory posits that individuals are generally more sensitive to losses during the decision-making process and therefore tend to take action to avoid potential losses (Kahneman and Tversky, 1979). Loss aversion plays a mediating role in the relationship between participation in extracurricular physical activity and sustained participation in extracurricular physical activity. Specifically, when adolescents experience benefits derived from exercise during the early stages of physical activity participation, discontinuing exercise may lead to a cognitive perception that “not exercising equals regression,” thereby motivating adolescents to continue engaging in physical activity in order to avoid losses. Accordingly, initial physical activity experiences not only shape individuals’ attitudes toward exercise but may also promote and sustain long-term participation in extracurricular physical activity through the psychological mechanism of loss aversion.

Drawing on self-determination theory and the trans-contextual model, when students perceive classroom need support from teachers, their intrinsic motivation is activated and subsequently transferred to extracurricular contexts, thereby promoting participation in extracurricular physical activity (T1). Subsequently, individuals gradually develop loss aversion during the exercise process, which further facilitates sustained participation in extracurricular physical activity. Accordingly, the following hypothesis is proposed:

H2: Adolescents’ positive perceptions of teachers’ need support can have a significant positive effect on their sustained participation in extracurricular physical activity through the sequential mediating effects of IMPE and IMECPA, as well as through loss aversion associated with early extracurricular physical activity (T1).

## 2 Materials and methods

### 2.1 Participants and procedures

This study was approved by the Academic Committee of Chongqing Jianzhu College (approval number: 20241001). Prior to data collection, the researchers provided detailed explanations of the research objectives and procedures to school administrators, physical education teachers, and all participants, and written informed consent was obtained from all participants. During the research process, participants were clearly informed that participation was entirely voluntary, that they could withdraw from the study at any time, and that withdrawal would not result in any adverse consequences.

The questionnaires were administered collectively during physical education classes, and a two-wave longitudinal follow-up was conducted starting in March 2025. The first wave of data collection was carried out in March 2025 (T1), primarily measuring students’ perceived need support, IMPE, IMECPA, perceived benefits of sports participation, loss aversion, and levels of extracurricular physical activity. The second wave of data collection was conducted in May 2025 (T2), during which students’ levels of extracurricular physical activity were measured again. The study design and data collection timeline are presented in Figure 2.

**FIGURE 2:**
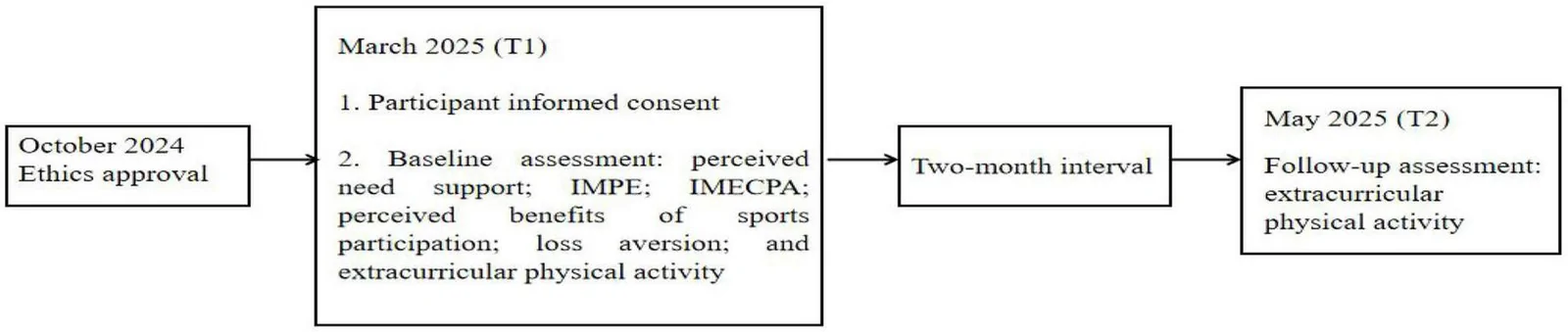
Study design and data collection timeline.

A total of 655 junior high school students were initially recruited for this study. After data screening, questionnaires with incomplete responses or invalid data were excluded, resulting in a final sample of 597 valid questionnaires and a response rate of 91.15%. In the final sample, there were 324 male students and 273 female students; 189 students were in Grade 7, 209 in Grade 8, and 199 in Grade 9. The mean age of the participants was 13.43 years (SD = 1.77).

### 2.2 Measures

All measurement instruments employed in this study were derived from well-established and validated scales published in leading academic journals and appropriately adapted to the physical education context. A 5-point Likert scale was used to measure extracurricular physical activity, while all other variables were assessed using 7-point Likert scales.

#### 2.2.1 Perceived need support scale

The perceived need support questionnaire comprises three dimensions: autonomy support, competence support, and relatedness support. The autonomy support dimension was adapted from the Health Care Climate Questionnaire developed by Williams and Deci (1996); Williams et al. (1996), with the item wording modified to reflect the physical education context. This dimension was used to assess whether physical education teachers provided students with autonomy in terms of choice and decision-making during instruction and included six items (e.g., “I feel that my physical education teacher provides us with many opportunities to make choices in class”). The Cronbach’s α for this dimension was 0.943. The competence support and relatedness support dimensions were adapted from the Competence and Relatedness Support Scale developed by Standage et al. (2005), with the item wording modified to reflect the physical education context. The competence support dimension assessed students’ perceived competence support in physical education classes and included four items (e.g., “The physical education teacher makes us feel capable of completing the activities in class”), with a Cronbach’s α of 0.903. The relatedness support dimension assessed students’ perceived relatedness support in physical education classes and included five items (e.g., “The physical education teacher treats us kindly”), with a Cronbach’s α of 0.946.

#### 2.2.2 Intrinsic motivation in physical education scale

Intrinsic motivation in physical education was measured using the intrinsic motivation subscale of the Physical Education Motivation Questionnaire, also known as the Perceived Reasons Scale, originally developed by Goudas et al. (1994) and later revised by Chung et al. (2014). This subscale was designed to assess students’ motivation to participate in physical education classes driven by intrinsic interest and enjoyment and included three items (e.g., “Physical education classes are interesting”). In the present study, the Cronbach’s α for this subscale was 0.818.

#### 2.2.3 Intrinsic motivation in extracurricular physical activity scale

Intrinsic motivation in extracurricular physical activity was measured using the intrinsic motivation subscale of the Behavioral Regulation in Exercise Questionnaire, as revised by Liu et al. (2015). This subscale was designed to assess students’ motivation to engage in extracurricular physical activity driven by interest and enjoyment and included four items (e.g., “I find extracurricular physical exercise enjoyable”). In the present study, the Cronbach’s α for this subscale was 0.946.

#### 2.2.4 Perceived benefits of sports participation scale

Perceived benefits of sports participation were measured using the exercise benefits dimension of the Exercise Benefits and Barriers Scale, originally developed by Ishii et al. (2009) and revised by Ying and Li (2022), with modifications to suit the physical education classroom context. This scale was used to assess adolescents’ perceived positive gains derived from participation in extracurricular physical activity and included 10 items (e.g., “Extracurricular physical exercise helps relieve stress and relax the mind”). In the present study, the Cronbach’s α for this scale was 0.946.

#### 2.2.5 Loss aversion in physical activity scale

Loss aversion in sports was measured using the Loss Aversion Scale developed by Li et al. (2021), with item selection and revision informed by the validation study of Cabedo-Peris et al. (2024), and further adapted to the physical activity context. This scale was used to assess adolescents’ loss aversion in sports-related decision-making and included six items (e.g., “When deciding whether to exercise, the first thing I think about is whether not exercising will cause me to regress”). In the present study, the Cronbach’s α for this scale was 0.906.

#### 2.2.6 Extracurricular physical activity scale

Extracurricular physical activity was measured using the Physical Activity Rating Scale-3 (PARS-3) developed by Liang (1994), with appropriate adaptations to the context of extracurricular physical activity. This scale was used to assess adolescents’ levels of physical activity during their leisure time and comprises three dimensions: exercise intensity, exercise duration, and exercise frequency, with a total of three items (e.g., “What is the intensity of your physical exercise during your leisure time?”). In the present study, the Cronbach’s α values for T1 and T2 were 0.857 and 0.842, respectively.

### 2.3 Statistical analysis

Before evaluating the structural equation model (SEM), a confirmatory factor analysis (CFA) was first conducted to examine the convergent and discriminant validity of all measurement scales. Given the relatively large number of items in the perceived need support scale, the item parceling approach was applied following the recommendation of Wu and Wen (2011). Specifically, items were parceled according to their corresponding subdimensions, and each dimension was used as an indicator of the latent variable to simplify the measurement model.

Subsequently, descriptive statistics and correlation analyses were performed using SPSS 25.0 to provide a preliminary understanding of the variables’ basic characteristics and their interrelationships. Considering that all variables in this study were self-reported, the data may have been influenced by social desirability effects, potentially leading to common method bias. Therefore, Harman’s single-factor test was employed to assess the presence of common method bias.

On this basis, the structural equation model was tested using AMOS 24.0 with the maximum likelihood estimation method. To estimate the mediating effects, the product-of-coefficients approach proposed by Hayes (2013) was adopted, in combination with the Monte Carlo bootstrapping method with bias correction developed by Preacher and Selig (2012). The significance of the mediating effects was determined based on whether the bias-corrected 95% confidence interval (CI) excluded zero; a CI not containing zero indicated a statistically significant mediation effect.

## 3 Results

### 3.1 Test for common method bias

This study used Harman’s single-factor test to examine common method bias. Excluding demographic variables, all other items were subjected to an unrotated exploratory factor analysis with a single factor. The results showed that six factors had initial eigenvalues greater than 1, and the first factor explained 37.455% of the total variance, which was lower than the commonly accepted threshold of 40%. Therefore, the data in this study did not indicate any serious common method bias.

### 3.2 Confirmatory factor analysis

Before evaluating the structural equation model, it was first necessary to examine the validity and adequacy of the measurement model. Only when the measurement model achieved an acceptable level of fit were the results of the complete structural equation model further reported. In the confirmatory factor analysis, the item reduction procedure followed the methodological recommendations of Kline (2005). In the perceived benefits of sports participation scale, the standardized factor loadings of PBSP9 and PBSP10 were below the acceptable threshold and were therefore deleted. As shown in Table 1, the factor loadings ranged from 0.659 to 0.933, the composite reliability (CR) ranged from 0.840 to 0.941, and the average variance extracted (AVE) ranged from 0.546 to 0.842. All these results met the criteria proposed by Hair et al. (1998): (1) factor loadings greater than 0.50; (2) composite reliability greater than 0.60; and (3) average variance extracted greater than 0.50. Therefore, all seven dimensions demonstrated good reliability and convergent validity.

**TABLE 1:** Summary of confirmatory factor analysis.

Latent variable	Observed variable	Unstd.	SE	*z*-value	*p*	Std.	SMC	CR	AVE
PNS	NA	1.000				0.911	0.830	0.941	0.842
	NC	1.017	0.027	37.667	***	0.933	0.870		
	NR	0.879	0.025	35.160	***	0.908	0.824		
IMPE	IMPE1	1.000				0.851	0.724	0.840	0.638
	IMPE2	0.793	0.042	18.881	***	0.848	0.719		
	IMPE3	0.934	0.055	16.982	***	0.686	0.471		
IMECPA	IMECPA1	1.000				0.900	0.810	0.905	0.706
	IMECPA2	0.891	0.036	24.750	***	0.801	0.642		
	IMECPA3	0.977	0.037	26.405	***	0.834	0.696		
	IMECPA4	0.869	0.033	26.333	***	0.822	0.676		
T1ECPA	T1ECPA1	1.000				0.836	0.699	0.857	0.667
	T1ECPA2	0.999	0.049	20.388	***	0.820	0.672		
	T1ECPA3	0.936	0.047	19.915	***	0.794	0.630		
PBSP	PBSP1	1.000				0.771	0.594	0.905	0.546
	PBSP2	1.004	0.052	19.308	***	0.758	0.575		
	PBSP3	1.063	0.056	18.982	***	0.748	0.560		
	PBSP4	1.011	0.054	18.722	***	0.744	0.554		
	PBSP5	1.059	0.054	19.611	***	0.772	0.596		
	PBSP6	0.942	0.053	17.774	***	0.709	0.503		
	PBSP7	1.056	0.057	18.526	***	0.741	0.549		
	PBSP8	0.811	0.050	16.220	***	0.659	0.434		
LA	LA1	1.000				0.761	0.579	0.907	0.619
	LA2	1.041	0.049	21.245	***	0.833	0.694		
	LA3	0.961	0.051	18.843	***	0.750	0.563		
	LA4	0.951	0.052	18.288	***	0.733	0.537		
	LA5	1.033	0.049	21.082	***	0.834	0.696		
	LA6	1.034	0.051	20.275	***	0.802	0.643		
T2ECPA	T2ECPA1	1.000				0.808	0.653	0.843	0.641
	T2ECPA2	0.980	0.054	18.148	***	0.774	0.599		
	T2ECPA3	1.012	0.054	18.741	***	0.819	0.671		

PNS, perceived need support; NA, need for autonomy; NC, need for competence; NR, need for relatedness; IMPE, intrinsic motivation in physical education; IMECPA, intrinsic motivation in extracurricular physical activity; T1ECPA, extracurricular physical activity (T1); PBSP, perceived benefits of sports participation; LA, loss aversion; T2ECPA, extracurricular physical activity (T2).

***p < 0.001.

### 3.3 Descriptive statistics and correlations

As shown in Table 2, the absolute values of skewness ranged from 0.049 to 1.403 (below the threshold of 2), and the absolute values of kurtosis ranged from 0.120 to 3.178 (below the threshold of 8), meeting the criteria proposed by Zhang et al. (2020). Therefore, the data in this study did not exhibit serious problems of skewness or kurtosis, and the overall distribution was approximately normal. In addition, the AVE method was used to test discriminant validity, which requires that the square root of each construct’s AVE be greater than its correlations with other constructs, indicating satisfactory discriminant validity among variables. As shown in Table 2, the square root of the AVE for each construct (on the diagonal) was greater than its corresponding correlation coefficients, demonstrating good discriminant validity among all latent variables and further confirming the reliability and validity of the measurement instruments used in this study.

**TABLE 2:** Descriptive statistics, correlations and discriminant validity.

Variables	M	SD	Skew	Kurtosis	PNS	IMPE	IMECPA	T1 ECPA	PBSP	LA	T2 ECPA
PNS	6.078	1.152	−1.198	0.433	***0.918***	***0.799***	***0.840***	***0.817***	***0.739***	***0.787***	***0.801***
IMPE	6.103	1.191	−1.403	1.410	0.803**						
IMECPA	5.492	1.120	−0.567	−0.120	0.237**	0.235**					
T1ECPA	2.749	0.878	0.049	−0.448	0.260**	0.278**	0.428**				
PBSP	5.712	0.941	−1.264	3.178	0.514**	0.533**	0.459**	0.379**			
LA	4.461	1.145	−0.311	−0.138	0.171**	0.155**	0.394**	0.419**	0.389**		
T2ECPA	3.190	0.884	−0.087	−0.513	0.286**	0.280**	0.433**	0.453**	0.459**	0.426**	

Bold italic values represent the square roots of AVE. **p < 0.01. Abbreviations are the same as in Table 1.

### 3.4 Structural equation model path analysis

Since the sample size in this study exceeded 200, which could lead to inflated chi-square values and consequently lower model fit indices, the correction procedure was applied (Su and Xu, 2017). After model fit correction using the Bollen-Stine Bootstrap procedure, the results showed: x^2^ = 975.811, x^2^/df = 2.452, GFI = 0.919, AGFI = 0.903, CFI = 0.950, IFI = 0.951, TLI = 0.946, and RMSEA = 0.049. All indices met the recommended standards, indicating that the model demonstrated a good overall fit and was acceptable for further analysis. The standardized path estimates of the chained mediation model are presented in Figure 3.

**FIGURE 3:**
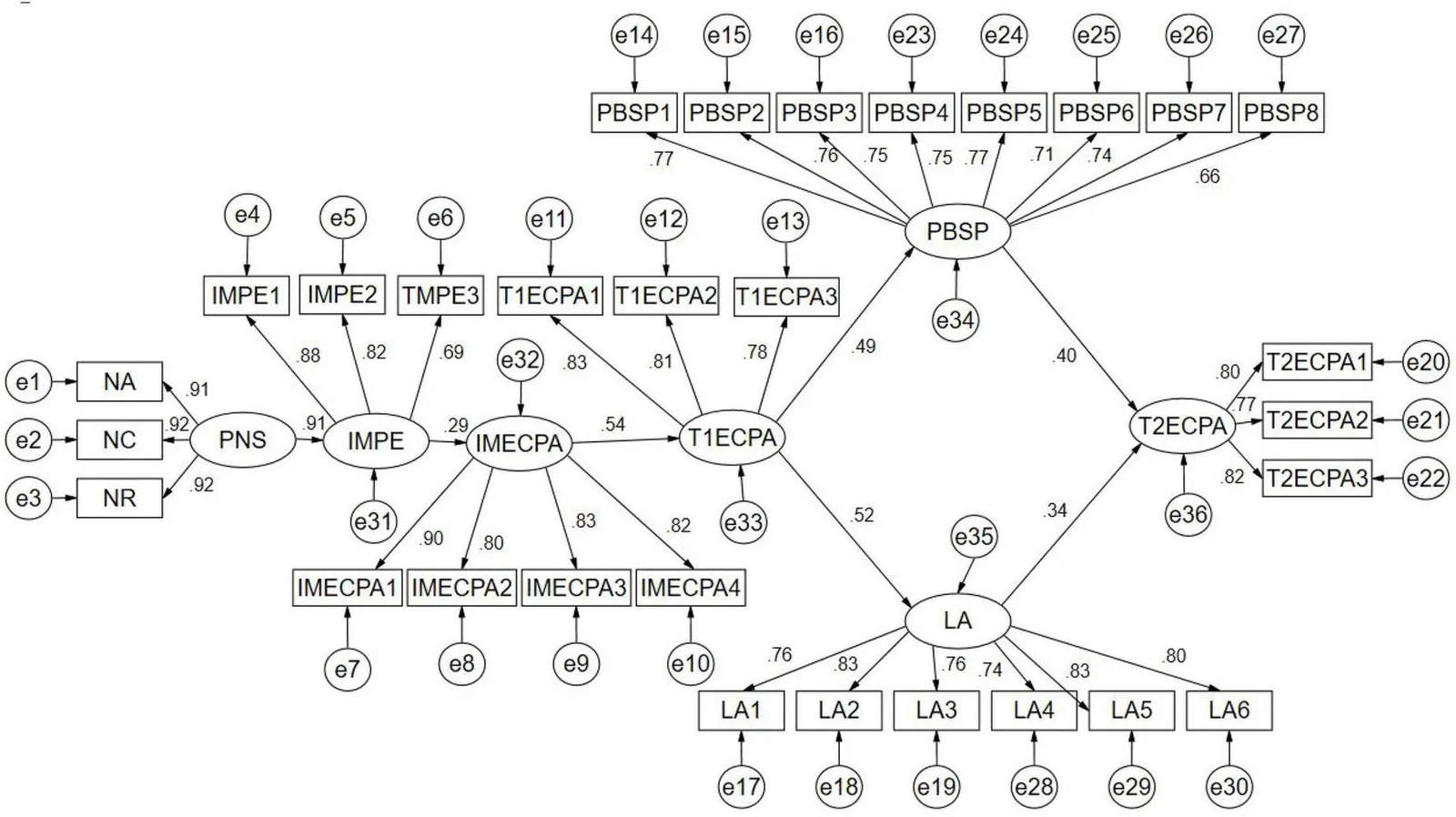
Diagram of the chained mediation model. Abbreviations are the same as in Table 1.

Regarding the path coefficients, perceived need support positively influenced IMPE (γ = 0.967, *p* < 0.001); IMPE positively affected IMECPA (γ = 0.269, *p* < 0.001). IMECPA positively influenced extracurricular physical activity (T1) (γ = 0.389, *p* < 0.001). Extracurricular physical activity (T1) positively affected perceived benefits of sports participation (γ = 0.684, *p* < 0.001) and loss aversion (γ = 0.539, *p* < 0.001). Perceived benefits of sports participation positively influenced extracurricular physical activity (T2) (γ = 0.359, *p* < 0.001); and loss aversion also had a significant positive effect on extracurricular physical activity (T2) (γ = 0.250, *p* < 0.001). The detailed path coefficients are presented in Table 3.

**TABLE 3:** Structural model path coefficients.

Path	Point estimate	Product of coefficients		Bias-corrected 95% CI		*p*
	γ	σ	*Z*	Lower	Upper	
PNS→IMPE	0.967	0.036	26.861	0.879	1.049	<0.001
IMPE→IMECPA	0.269	0.041	6.561	0.186	0.355	<0.001
IMECPA→T1ECPA	0.389	0.032	12.156	0.323	0.456	<0.001
T1ECPA→PBSP	0.684	0.062	11.032	0.568	0.815	<0.001
T1ECPA→LA	0.539	0.052	10.365	0.434	0.650	<0.001
PBSP→T2ECPA	0.359	0.043	8.349	0.273	0.464	<0.001
LA→T2ECPA	0.250	0.035	7.143	0.178	0.330	<0.001

Abbreviations are the same as in Table 1.

### 3.5 Mediating effect analysis in the structural equation model

The mediating effects in the structural equation model were tested using the bias-corrected parametric Monte Carlo bootstrapping method based on 5,000 resamples. The results, as shown in Table 4, indicated that the mediating effect through the perceived benefits of sports participation path was significant (*Z* = 3.333, *p* < 0.001), and the mediating effect through the loss aversion path was also significant (*Z* = 4.250, *p* < 0.001).

**TABLE 4:** Mediation analysis of the structural equation model.

Hypothesis	Mediation Path	Point estimate	Product of coefficients		Bias-corrected 95% CI		*p*
		γ	σ	*Z*	Lower	Upper	
H1	PNS→IMPE→IMECPA→T1ECPA→PBSP→T2ECPA	0.020	0.006	3.333	0.011	0.033	<0.001
H2	PNS→IMPE→IMECPA→T1ECPA→LA→T2ECPA	0.017	0.004	4.250	0.010	0.028	<0.001

Abbreviations are the same as in Table 1.

## 4 Discussion

### 4.1 The promoting role of perceived benefits of sports participation

To elucidate the underlying mechanisms of adolescents’ sustained participation in extracurricular physical activity, this study drew on self-determination theory, the trans-contextual model, and prospect theory to construct a structural equation model focusing on intrinsic motivation, contextual transfer, and decision-making processes. The model included perceived classroom need support, IMPE, IMECPA, extracurricular physical activity at T1, perceived benefits of sports participation, loss aversion, and extracurricular physical activity at T2. The results indicated that adolescents’ perceived need support in physical education classes could promote their sustained participation in extracurricular physical activity through the sequential mediating effects of IMPE and IMECPA, as well as through the perceived benefits of sports participation derived from extracurricular physical activity. Therefore, Hypothesis 1 (H1) was supported.

Autonomy support, competence support, and relatedness support provided by physical education teachers during classroom instruction can satisfy students’ basic psychological needs, thereby enhancing IMPE. Intrinsic motivation developed in physical education classes can, through cross-contextual transfer, exert a positive influence on IMECPA. This finding is consistent with previous research (Barkoukis et al., 2010; Vasconcellos et al., 2020; White et al., 2021). By providing autonomy, competence, and relatedness support in the classroom, physical education teachers help to create a supportive instructional climate, reduce students’ psychological pressure and perceived external control, and promote the formation and development of intrinsic motivation. On this basis, compared with support for a single psychological need, the synergistic satisfaction of all three basic psychological needs is more conducive to enhancing students’ intrinsic motivation (Deci et al., 1994; Deci and Ryan, 2000). Based on the trans-contextual model, intrinsic motivation formed in physical education classes can be extended to the context of extracurricular physical activity, leading students to exhibit stronger interest, engagement, and autonomous participation in extracurricular physical activity. Specifically, intrinsic motivation developed in physical education classes encourages students to actively seek opportunities for relevant physical activities outside the classroom and to participate in them voluntarily (Hagger and Chatzisarantis, 2007).

Extracurricular physical activity at T1 can significantly predict the level of participation in extracurricular physical activity at T2 through the perceived benefits of sports participation. Perceived benefits of sports participation constitute an important psychological foundation for maintaining adolescents’ engagement in physical activity, and their role can be understood from two perspectives: positive experiences and exercise-related benefits. From the perspective of positive experiences, the continuous emergence of positive emotional experiences during physical activity–such as interest, enjoyment, and intrinsic satisfaction–renders exercise behavior inherently attractive, thereby strengthening individuals’ willingness to continue participating. In this process, exercise enjoyment and fun play a crucial role. When adolescents are able to consistently experience pleasure and enjoyment derived from extracurricular physical activity, their emotional engagement is strengthened, which in turn promotes long-term participation. Conversely, when the experiential quality of exercise is poor, adolescents’ evaluations of physical activity and their intentions to continue participating tend to decline markedly. Previous studies have demonstrated that both exercise enjoyment and fun significantly predict future physical activity participation among children and adolescents and serve as important psychological foundations for sustained engagement (Cairney et al., 2012; Crane and Temple, 2015; Visek et al., 2015; Burns et al., 2017; Fierro-Suero et al., 2022). From the perspective of exercise-related benefits, extracurricular physical activity not only enhances adolescents’ physical fitness but also improves their emotional states and strengthens their sense of belonging through peer interaction (Eime et al., 2013). When adolescents perceive a positive association between their exercise investment and the resulting physical and psychological benefits, such benefit perceptions are transformed into psychological momentum that supports sustained participation. In summary, through the dual functions of positive experiences and exercise-related benefits, perceived benefits of sports participation reinforce adolescents’ value recognition of extracurricular physical activity and their intentions to engage in it on a sustained basis.

### 4.2 The promoting role of loss aversion

The results indicated that adolescents’ perceived need support in physical education classes promoted their sustained participation in extracurricular physical activity through the sequential mediating effects of IMPE and IMECPA, as well as through loss aversion elicited by participation in extracurricular physical activity. Therefore, Hypothesis 2 (H2) was supported. Need support in physical education can satisfy students’ needs for autonomy, competence, and relatedness, thereby fostering their intrinsic motivation in physical education. This motivation can subsequently transfer to extracurricular contexts, strengthening students’ interest in and willingness to voluntarily participate in extracurricular physical activity (Hagger et al., 2003; Barkoukis et al., 2010).

In the decision-making process of sports participation, individuals are driven not only by positive incentives but also exhibit sensitivity to and avoidance of potential losses (Kahneman and Tversky, 1979). Extracurricular physical activity at T1 can significantly predict participation in extracurricular physical activity at T2 through loss aversion. For adolescents, this loss-avoidance tendency is particularly important in sustaining their participation in extracurricular physical activity. When adolescents engage continuously in extracurricular physical activity at T1 and obtain positive outcomes such as improvements in physical fitness and body shape, these benefits tend to be internalized as “acquired gains.” Once exercise behavior is interrupted or participation decreases, adolescents often worry that the physical achievements they have previously attained may be lost. This anxiety and concern about “losing existing gains” become an important psychological mechanism motivating continued participation in physical activity. To avoid such outcomes, adolescents are more inclined to maintain or even increase their level of exercise. In addition, loss aversion can influence individuals through processes of social comparison. When individuals obtain less than others, even if the outcome is objectively positive, they may still experience a sense of relative loss. When adolescents observe that their peers continue to participate in extracurricular physical activity while they themselves fail to persist, they are likely to experience a sense of relative disadvantage. Such social comparison further strengthens individuals’ motivation to reengage in or persist with extracurricular physical activity (Diel et al., 2021; Spray et al., 2023). In summary, loss aversion not only manifests as concern over the loss of previously acquired benefits but also operates through feelings of relative loss arising from social comparison, thereby promoting sustained participation in extracurricular physical activity.

## 5 Conclusion

Adolescents’ perceived need support in physical education classes can promote their sustained participation in extracurricular physical activity through the sequential effects of IMPE and IMECPA, as well as through the perceived benefits of sports participation and loss aversion elicited by extracurricular physical activity.

Based on the above findings, the present study proposes the following practical implications. First, physical education teachers should promote the satisfaction of students’ need for autonomy, need for competence, and need for relatedness by optimizing instructional practices in the classroom. Specifically, teachers can provide students with appropriate opportunities for choice during instructional activities, design tasks with suitable levels of challenge, and strengthen emotional support in teacher-student interactions, thereby fostering a supportive and autonomy-oriented classroom climate and enhancing students’ classroom engagement. Second, students should be guided to attach greater importance to the roles of perceived benefits of sports participation and loss aversion in sustaining physical activity. By strengthening their perceived benefits of sports participation and appropriately addressing loss aversion, students’ willingness to engage in physical activity on a sustained basis can be effectively enhanced, thereby facilitating the development of long-term exercise habits.

## 6 Limitations and future directions

This study has several limitations that should be acknowledged in future research. First, the variables were primarily derived from students’ self-reports, which may be influenced by social desirability effects and common method bias. Future studies should incorporate objective indicators (e.g., exercise data recorded by wearable devices) to improve measurement objectivity (Podsakoff et al., 2024). Second, the study sample was relatively localized, including only junior high school students from certain regions, which limits the generalizability of the findings to adolescents from different areas, grade levels, or cultural backgrounds. Subsequent research could expand the scope by conducting cross-regional and cross-cultural validation to examine the stability and universality of the proposed model paths. Finally, this study adopted a two-wave longitudinal design with a relatively short time interval, which primarily revealed the short-term relationships between motivational transfer and behavioral maintenance but did not capture the dynamic changes in physical activity over a longer period. Future research should extend the tracking period or introduce multi-wave measurements to more comprehensively reveal the long-term processes and psychological mechanisms underlying adolescents’ sustained physical activity (Rijnhart et al., 2022).

## Data Availability

The raw data supporting the conclusions of this article will be made available by the authors, without undue reservation.
